# Cohort Profile: The North East London Diabetes Study (NELDS)

**DOI:** 10.1093/ije/dyag117

**Published:** 2026-08-05

**Authors:** Abraham Olvera-Barrios, Alasdair N Warwick, Matilda Pitt, Zeinab Schaefer, Schanhave Santhirasekaran, Ryan Chambers, Louis Bolter, John Anderson, Jiri Fajtl, Sarah Barman, Reecha Sofat, Aroon Hingorani, Catherine Egan, Adnan Tufail, Alicja R Rudnicka, Christopher G Owen, the ARIAS Research Group, Nayani G Adhikari, John Anderson, Sarah Barman, Louis Bolter, Ryan Chambers, Lakshmi Chandrasekaran, Umar Chaudhry, Catherine Egan, Jiri Fajtl, Aaron Lee, Abraham Olvera-Barrios, Christopher G Owen, Paolo Remagnino, Alicja R Rudnicka, Royce D Shakespeare, Adnan Tufail, Charlotte Wahlich, Alasdair N Warwick, Roshan Welikala

**Affiliations:** NIHR Biomedical Research Centre, Moorfields Eye Hospital NHS Foundation Trust & University College London Institute of Ophthalmology, London, United Kingdom; NIHR Biomedical Research Centre, Moorfields Eye Hospital NHS Foundation Trust & University College London Institute of Ophthalmology, London, United Kingdom; Institute of Cardiovascular Science, University College London, London, United Kingdom; Department of Population Health and Policy, City St George’s, University of London, London, United Kingdom; Department of Population Health and Policy, City St George’s, University of London, London, United Kingdom; Department of Population Health and Policy, City St George’s, University of London, London, United Kingdom; Diabetes, Homerton Healthcare NHS Foundation Trust, London, United Kingdom; Diabetes, Homerton Healthcare NHS Foundation Trust, London, United Kingdom; Diabetes, Homerton Healthcare NHS Foundation Trust, London, United Kingdom; School of Computer Science and Mathematics, Kingston University, London, United Kingdom; School of Computer Science and Mathematics, Kingston University, London, United Kingdom; Department of Pharmacology and Therapeutics, University of Liverpool, Liverpool, United Kingdom; Institute of Cardiovascular Science, University College London, London, United Kingdom; NIHR Biomedical Research Centre, Moorfields Eye Hospital NHS Foundation Trust & University College London Institute of Ophthalmology, London, United Kingdom; NIHR Biomedical Research Centre, Moorfields Eye Hospital NHS Foundation Trust & University College London Institute of Ophthalmology, London, United Kingdom; Department of Population Health and Policy, City St George’s, University of London, London, United Kingdom; Department of Population Health and Policy, City St George’s, University of London, London, United Kingdom

Key Features

- The North East London Diabetes Study (NELDS) was established to investigate the epidemiology of diabetes-related outcomes and to support the development of equitable predictive models.
- Based in North East London, England, the NELDS includes people with diabetes (92% type 2) registered with the Diabetic Eye Screening Programme (DESP) between January 2012 and December 2021. The cohort comprises 178 536 individuals aged ≥12 years at baseline (median age 57 years; interquartile range 47–68). Ethnic diversity is substantial, with 36% White, 34% South Asian, and 16% Black participants. By December 2021, 15% had died and 9.8% had moved out of the area.
- The DESP dataset includes 5.5 million colour fundus photographs, standardized diabetic retinopathy grading, and socio-demographic information spanning 1 074 985 person-years of follow-up. These data are linked to primary and secondary care electronic health records (EHRs) containing clinical measurements, medication records, and comorbidity information. Prospective data collection is ongoing.
- The scale, diversity, and unique integration of longitudinal ophthalmic imaging with linked EHR data of the NELDS make it well suited for developing accurate, equitable predictive models to inform personalized care and improve the prevention of diabetes complications.
- Enquiries about collaboration and data access should be directed to the corresponding author.

## Why was the cohort set up?

Diabetes is a growing public health problem affecting 1 in 10 adults globally [1]. The number of people with diabetes worldwide increased from 151 million in 2000 to 589 million in 2024 and is projected to rise to 853 million by 2050 [2]. About half remain undiagnosed [3]. Furthermore, an estimated 635 million adults have impaired fasting glucose or impaired glucose tolerance, which progresses to type 2 diabetes at a rate of 5%–10% per year [4–6]. In the UK, an estimated 5 million people have diabetes, costing the National Health Service (NHS) ∼6% of the health budget [7, 8]. In 2024 alone, there were an estimated 3.4 million diabetes-related deaths worldwide, with 37% occurring in the working-age population [2].

The early identification of individuals at risk of complications is crucial for targeted intervention. Studies have sought to estimate the risk of major complications, including all-cause mortality, myocardial infarction, stroke, renal disease, and sight-threatening diabetic retinopathy (STDR) [9–12]. Diabetic retinopathy (DR)—a major microvascular complication of diabetes and leading cause of blindness in the working-age population [13]—provides a window to systemic health, with studies demonstrating the utility of incorporating DR grading into predictive models for adverse health outcomes [14–17]. However, >80% of ophthalmic imaging datasets lack socio-demographic characteristics and population diversity [18]; ethnic minorities and people with type 1 diabetes are typically under-represented and model translation is lacking [19].

The North East London Diabetes Study (NELDS) is a large cohort study of people registered with the North East London Diabetic Eye Screening Programme (NEL DESP), designed to harness routinely collected electronic health record (EHR) data to improve prediction and early intervention for complications in people with diabetes. Data from the NEL DESP, funded through the NHS public health budget, are available from 2012 following the implementation of the region’s current database system. The study combines longitudinal socio-demographic data, colour fundus photographs, manually graded DR severities, and screening outcomes with linked primary and secondary care EHRs from an ethnically diverse cohort. This provides detailed phenotypic information across a wide range of biomarkers and health outcomes, facilitating the development of strategies to enhance care for people with diabetes.

## Who is in the cohort?

The NELDS is a cohort study of 178 536 people diagnosed with diabetes aged ≥12 years who were registered for population diabetic eye screening in the NEL DESP between 3 January 2012 and 31 December 2021. Prospective data collection is ongoing. Ethics approval did not require individual consent, as the study relied solely on retrospective, anonymized data (further details in the ‘Ethics approval’ section).

The uptake of DR screening in the UK is high (consistently >80% annual uptake) [20], driven by incentives under the Quality and Outcomes Framework for GPs to refer those with newly diagnosed diabetes for eye screening. This ensures a comprehensive and systematically collected ophthalmic dataset for the NELDS. Further details for the national and NEL DESP are provided in the Supplementary methods. While data are not available for non-participants (eligible individuals with diabetes who did not attend) or the general population of individuals with diabetes, the strong representation of high-risk groups in the NEL DESP cohort provides valuable insights.

Table 1 summarizes key socio-demographic characteristics for the full cohort, as well as by diabetes subtype. The median age at baseline DESP appointment was 57 years (interquartile range 47–68) and 46% were female. The majority of individuals (92%) had type 2 diabetes, while 4.6% had type 1. The remainder (grouped together as ‘Other’ in Table 1) were recorded as Maturity Onset Diabetes of the Young (MODY) (*n* = 116), Other (*n* = 287), or Unknown (*n* = 6592). Individuals with type 1 diabetes were considerably younger at diagnosis (median age 21 years), with a longer median diabetes duration (8 years) compared with the overall cohort.

**Table 1:** Summary of socio-demographic factors at baseline DESP visit. Results are presented for the full cohort (shaded in grey) and by diabetes subtype.

Full cohort	Diabetes subtype			
Characteristic	*N* = 178 536	Type 2 (*N* = 163 393)	Type 1 (*N* = 8148)	**Other** ^a^ **(*N* = 6995)**
**Age (years)**				
Median (Q1, Q3)	57 (47, 68)	57 (48, 68)	32 (23, 46)	63 (49, 76)
**Sex [*n* (%)]**				
Female	81 714 (46)	74 889 (46)	3627 (45)	3198 (46)
Male	96 796 (54)	88 483 (54)	4520 (55)	3793 (54)
Unknown	26 (<0.1)	21 (<0.1)	1 (<0.1)	4 (<0.1)
**Diabetes duration (years)**				
Median (Q1, Q3)	2 (0, 8)	2 (0, 8)	8 (1, 17)	1 (0, 5)
*N* missing (%)	4259 (2.4)	997 (0.6)	79 (1.0)	3183 (46)
**Age at diabetes diagnosis (years)**				
Median (Q1, Q3)	52 (42, 62)	52 (43, 62)	21 (12, 33)	54 (41, 66)
*N* missing (%)	4259 (2.4)	997 (0.6)	79 (1.0)	3183 (46)
**Died [*n* (%)]**	26 547 (15)	23 476 (14)	666 (8.2)	2405 (34)
**Age at death (years)**				
Median (Q1, Q3)	79 (70, 86)	79 (70, 86)	69 (55, 80)	80 (71, 86)
**Ethnicity [*n* (%)]**				
White	64 592 (36)	56 533 (35)	5093 (63)	2966 (42)
South Asian	61 202 (34)	58 880 (36)	1109 (14)	1213 (17)
Black	29 103 (16)	27 269 (17)	960 (12)	874 (12)
Any other Asian	9838 (5.5)	9457 (5.8)	190 (2.3)	191 (2.7)
Other	5394 (3.0)	4974 (3.0)	255 (3.1)	165 (2.4)
Unknown	4833 (2.7)	3073 (1.9)	303 (3.7)	1457 (21)
Mixed	2384 (1.3)	2093 (1.3)	197 (2.4)	94 (1.3)
Chinese	1190 (0.7)	1114 (0.7)	41 (0.5)	35 (0.5)
**IMD quintile [*n* (%)]**				
1	61 097 (34)	56 737 (35)	2664 (33)	1696 (24)
2	58 135 (33)	54 042 (33)	2547 (31)	1546 (22)
3	30 395 (17)	28 300 (17)	1285 (16)	810 (12)
4	13 280 (7.4)	12 163 (7.4)	686 (8.4)	431 (6.2)
5	4281 (2.4)	3801 (2.3)	265 (3.3)	215 (3.1)
Unknown	11 348 (6.4)	8350 (5.1)	701 (8.6)	2297 (33)
**Smoking history [*n* (%)]**				
Ever smoked	52 758 (30)	48 811 (30)	2378 (29)	1569 (22)
Never smoked	100 000 (56)	93 279 (57)	4336 (53)	2385 (34)
Unknown	25 778 (14)	21 303 (13)	1434 (18)	3041 (43)
**Family history of CVD [*n* (%)]**	66 315 (37)	61 794 (38)	2228 (27)	2293 (33)

a ‘Other’ includes individuals for whom the type of diabetes was recorded as ‘Maturity Onset of the Young’, ‘Other’, or ‘Unknown’.

Findings are presented as median (interquartile range) for continuous variables and number (%) for categorical variables.

CVD, cardiovascular disease; IMD, Index of Multiple Deprivation.

The NELDS cohort demonstrates considerable ethnic diversity, with 36% of individuals self-reporting as being of White ethnicity, 34% as South Asian, and 16% as Black. The majority (67%) of individuals resided in the two most deprived Index of Multiple Deprivation (IMD) quintiles. Supplementary Table 1 further highlights ethnic disparities within deprivation, with Black (46% in Q1) and South Asian (36% in Q1) groups having a higher representation in the most deprived quintile of IMD compared with White individuals (29% in Q1). South Asian individuals were diagnosed with diabetes at a younger median age (47 years) than White individuals (56 years).

## How often have they been followed up?

**Figure 1:**
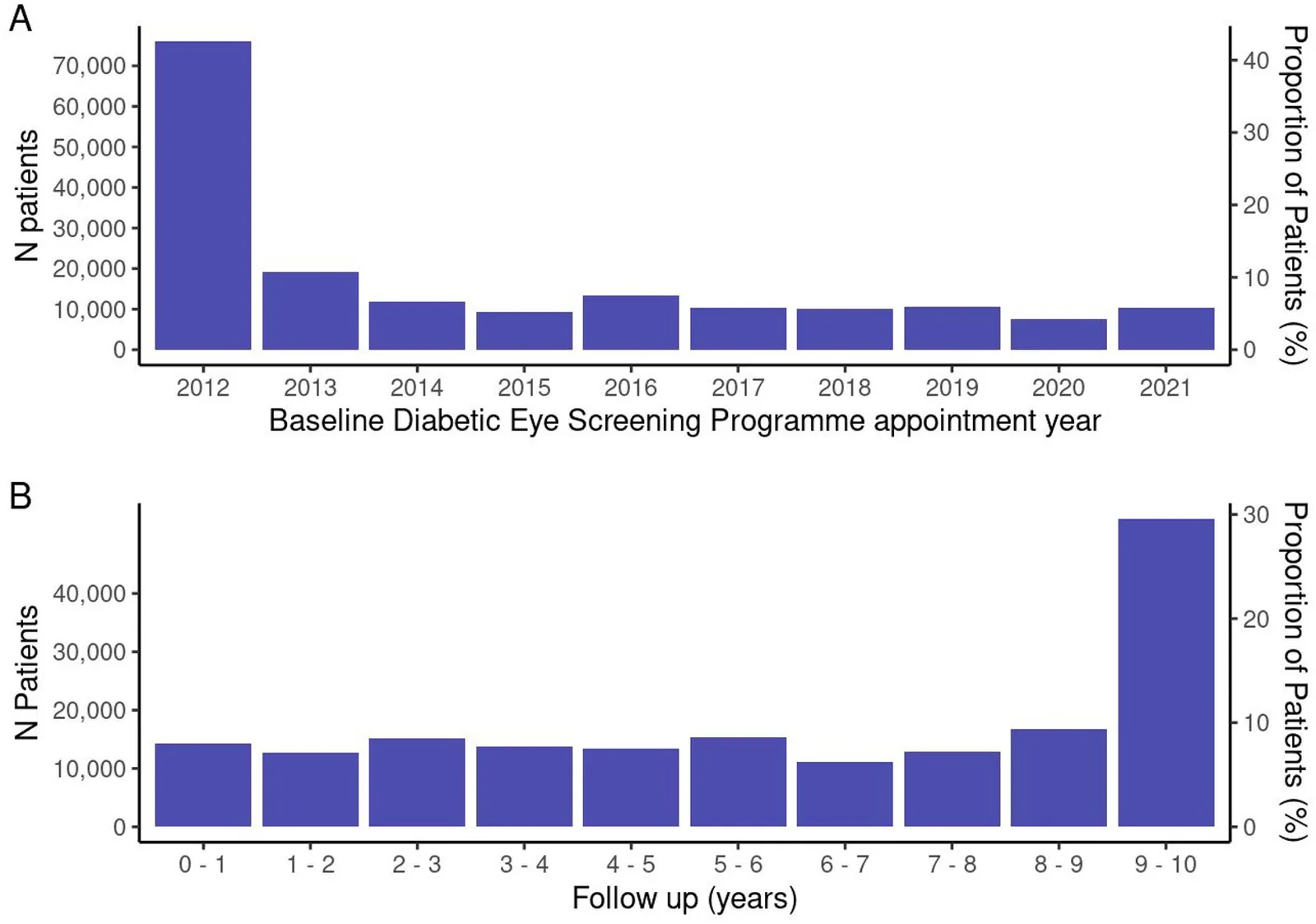
Distribution of patients in the DESP cohort. (a) Number of patients by year of baseline DESP appointment, showing recruitment patterns over time. (b) Distribution of follow-up duration in years, indicating the range of longitudinal data available for analysis.

Participants undergo annual or more frequent DR screening as part of routine NHS care, with recent policy changes allowing biennial screening for low-risk individuals [21]. During the study period (2012–21), the NEL DESP dataset includes a total of 1 130 390 appointments and >5.5 million retinal images spanning >1 million person-years. The median follow-up duration was 6.4 years (interquartile range [IQR] 3.2, 9.2), with 61.1% of patients having ≥5 years of follow-up from their baseline DESP appointment (Fig. 1).

By the end of 2021, 15% of individuals in the cohort had died, with a median age at death of 79 years (IQR 70, 86). 9.8% had moved out of the area. Supplementary Table 2 compares the baseline characteristics of the 43 972 individuals permanently lost to follow-up (due to death or moving out of area) with the continuing cohort. These individuals were older with longer diabetes duration and more likely to have missing data.

## What has been measured?

The NEL DESP dataset includes colour fundus photographs, standardized grading for DR (Supplementary methods), and socio-demographic information. Linked EHR data were available for 99.5% of individuals, capturing all historic and contemporary primary and secondary care records, providing clinical measurements, medication details, and comorbidity data. The methodology for defining clinical measurements and diabetes-related comorbidities from EHR data are detailed in the Supplementary methods—‘Characterization at baseline’. The complete final list of included SNOMED CT codes and numbers of EHR entries is available in the Supplementary Excel file.

## What has it found?

### Clinical measurements

High completeness of linked primary care data for key clinical measurements was achieved, with >95% of individuals having at least one measurement for haemoglobin A1C (HbA1c), total cholesterol, systolic blood pressure, estimated glomerular filtration rate (eGFR), and body mass index (BMI) (Supplementary Table 3). Detailed clinical characteristics are presented in Table 2 and Supplementary Table 4. Individuals with type 1 diabetes had a higher median HbA1c value (69 mmol/mol) compared with those with type 2 (53 mmol/mol). Notable ethnic differences were observed in systolic blood pressure, BMI, and eGFR, with higher cardiovascular risk profiles in White and Black individuals compared with South Asian and Chinese groups (Supplementary Table 4).

**Table 2:** Summary of clinical measurements and medication history at baseline DESP visit. Results are presented for the full cohort (shaded in grey) and by diabetes subtype.

Full cohort	Diabetes subtype^a^			
Characteristic	*N* = 178 536	Type 2 (*N* = 163 393)	Type 1 (*N* = 8148)	Other (*N* = 6995)
**Systolic blood pressure (mmHg)**				
Median (Q1, Q3)	133 (124, 141)	133 (125, 141)	123 (115, 132)	134 (126, 142)
*N* missing (%)	4283 (2.4)	3244 (2.0)	712 (8.7)	327 (4.7)
**BMI (kg/m²)**				
Median (Q1, Q3)	29.2 (25.9, 33.5)	29.4 (26.2, 33.6)	24.6 (21.9, 28.3)	29.0 (25.5, 33.3)
*N* missing (%)	8999 (5.0)	7356 (4.5)	1072 (13)	571 (8.2)
**HbA1c (mmol/mol)**				
Median (Q1, Q3)	54 (48, 67)	53 (48, 66)	69 (56, 85)	50 (45, 64)
*N* missing (%)	30 437 (17)	26 224 (16)	2687 (33)	1526 (22)
**eGFR (ml/min/1.73 m²)**				
Median (Q1, Q3)	74 (60, 87)	74 (60, 87)	82 (60, 90)	60 (60, 80)
*N* missing (%)	37 733 (21)	32 524 (20)	3288 (40)	1921 (27)
**Total cholesterol (mmol/l)**				
Median (Q1, Q3)	4.59 (3.97, 5.27)	4.60 (3.97, 5.28)	4.50 (3.95, 5.13)	4.55 (3.93, 5.23)
*N* missing (%)	7455 (4.2)	5067 (3.1)	1756 (22)	632 (9.0)
**Retinopathy grade, worst [*n* (%)]**				
R0	127 312 (71)	118 477 (73)	4157 (51)	4678 (67)
R1	41 759 (23)	37 079 (23)	3081 (38)	1599 (23)
R2	3914 (2.2)	3195 (2.0)	442 (5.4)	277 (4.0)
R3	2270 (1.3)	1735 (1.1)	364 (4.5)	171 (2.4)
Unknown	3281 (1.8)	2907 (1.8)	104 (1.3)	270 (3.9)
**Maculopathy grade, worst [*n* (%)]**				
M0	163 274 (91)	150 222 (92)	6901 (85)	6151 (88)
M1	11 981 (6.7)	10 264 (6.3)	1143 (14)	574 (8.2)
Unknown	3281 (1.8)	2907 (1.8)	104 (1.3)	270 (3.9)
**Best VA (LogMAR)**				
Median (Q1, Q3)	0.00 (−0.10, 0.20)	0.00 (−0.10, 0.20)	0.00 (−0.10, 0.00)	0.00 (0.00, 0.20)
*N* missing (%)	1570 (0.9)	1352 (0.8)	58 (0.7)	160 (2.3)
**Anti-hypertension medication [*n* (%)]**	112 376 (63)	105 606 (65)	2291 (28)	4479 (64)
**Lipid-lowering medication [*n* (%)]**	109 465 (61)	103 208 (63)	2345 (29)	3912 (56)
**Non-insulin hypoglycaemic medication [*n* (%)]**	132 967 (74)	127 055 (78)	1981 (24)	3931 (56)
**On insulin [*n* (%)]**	27 973 (16)	19 393 (12)	7296 (90)	1284 (18)

a ‘Other’ includes individuals for whom the type of diabetes was recorded as ‘Maturity Onset of the Young’, ‘Other’, or ‘Unknown’.

Findings are presented as median (interquartile range) for continuous variables and number (%) for categorical variables. DR outcomes are recorded as per the UK National Screening Committee classification system (see Supplementary methods).

logMAR, logarithm of the minimum angle of resolution; VA, visual acuity.

Individuals with type 2 diabetes presented with higher median systolic blood pressure and BMI and lower median eGFR compared with those with type 1 diabetes. Correspondingly, a higher proportion of people with type 2 diabetes had commenced antihypertensive (65% vs 28% type 1), lipid-lowering (63% vs 29% type 1), and non-insulin hypoglycaemic (78% vs 24% type 1) medications (Table 2).

### Diabetic retinopathy

At baseline, 71% of the overall cohort had no detectable retinopathy (R0). However, as shown in Table 2, individuals with type 1 diabetes had a notably higher baseline prevalence of DR (48% R1 or worse) and diabetic maculopathy (14% M1) compared with those with type 2 diabetes (26% R1 or worse, 6.3% M1). A clear trend for worsening cardiometabolic risk factors, longer diabetes duration, and younger age at diagnosis was observed with increasing DR severity (Supplementary Tables 5 and 6). A total of 30 766 individuals developed referable DR (M1, R2, or R3) at some stage during the study period.

### Comorbidities and incident events

As shown in Table 3, hypertension was the most common prevalent comorbidity (90 456 cases), followed by renal failure (23 289 cases). Hypertension and renal failure also exhibited the highest incidence rates (41.5 and 20.8 per 1000 person-years, respectively). The incidence rates for coronary heart disease and stroke were 10.6 and 5.1 per 1000 person-years, respectively. As further illustrated in Supplementary Table 7, the incident rates for all comorbidities were observed to be higher in individuals with more severe baseline retinopathy. The majority of cases for all these conditions were ascertained from primary care records (Supplementary Figure 1).

**Table 3:** Prevalent cases of major comorbidities at baseline DESP visit and subsequent incident events. Incidence rates expressed per 1000 person-years.

Comorbidity	Prevalent cases	Crude prevalence (%)	**Age-standardized prevalence** ^a^ **[% (95% confidence interval)]**	*N* at risk	Incident cases	Crude incidence rate/1000 person-years	**Age-standardized incidence rate/1000 person-years** ^a^ **[*n* (95% confidence interval)]**
Coronary heart disease	21 350	12.0	3.8 (3.8, 3.9)	157 186	9680	10.6	3.8 (3.7, 3.9)
Foot ulcer	2625	1.5	1.0 (0.9, 1.2)	175 911	3463	3.3	2.1 (1.8, 2.4)
Hypertension	90 456	50.7	20.4 (20.2, 20.6)	88 080	17 911	41.5	20.9 (20.5, 21.4)
Peripheral artery disease	3289	1.8	0.6 (0.5, 0.6)	175 247	3580	3.4	1.4 (1.3, 1.5)
Renal failure	23 289	13.0	4.5 (4.4, 4.6)	155 247	18 168	20.8	8.2 (7.9, 8.5)
Stroke	5593	3.1	1.0 (1.0, 1.0)	172 943	5231	5.1	1.4 (1.4, 1.5)

a Age-standardized prevalence and incidence rates were calculated based on the World Health Organization 2000–2025 World Standard Population.

### Attendance patterns and socio-demographic disparities in screening uptake

Data from the NELDS have provided critical insights into the impact of socio-demographic factors on diabetic eye care, with a core focus on addressing health inequalities. Contrary to some prior assumptions, our analyses revealed higher odds ratios (ORs) of attending screening for South Asian [OR 1.16, 95% confidence interval (CI) 1.09–1.23], Chinese (OR 1.91, 95% CI 1.39–2.71), and Any other Asian (OR 1.30, 95% CI 1.17–1.45) individuals, with no significant difference observed for Black individuals compared with White [22]. Attendance was positively associated with socio-economic status (OR 1.06 per higher IMD quintile, 95% CI 1.03–1.08) [22] while younger age was associated with lower attendance (OR per decade increase 1.17, 95% CI 1.15–1.19) [22].

### Ethnic and age disparities in progression to STDR

Beyond the initial uptake, our research established contemporary incidence rates for progression to STDR and highlighted significant ethnic and age disparities [23]. Despite similar or better attendance at initial screening, Black individuals experienced a 57% increase in the hazards of STDR (HR 1.57, 95% CI 1.50–1.64) and South Asian individuals showed a 36% increase (HR 1.36, 95% CI 1.31–1.42) when compared with White participants. Moreover, younger age groups consistently showed higher hazards of STDR [23]. These findings collectively point to deeper systemic and/or biological factors contributing to health inequalities in disease progression, even when the initial screening engagement is comparable.

### Policy implications and equitable risk stratification

These disparities have direct policy implications. Following the introduction of biennial screening in England for low-risk individuals in October 2023 [21], simulations using NELDS data revealed that a blanket biennial approach would delay diagnosis for 56.3% of STDR and 43.6% of proliferative DR cases, disproportionately affecting Black, South Asian, and young individuals [24]. To counteract this, we developed a simplified risk score using age, diabetes duration, and ethnicity, which reduced delayed STDR diagnoses from 39% to 25% while achieving a 43% reduction in screening appointments compared with annual screening [25]. Extensive Patient and Public Involvement and Engagement activities [26] explored perceptions of artificial intelligence (AI)-assisted screening. While 24% of people living with diabetes and 37% of healthcare practitioners provided detailed comments, both groups largely viewed the introduction of AI as inevitable and beneficial for efficiency and cost savings [27]. However, concerns were raised regarding job losses, data security, dehumanization of the patient–practitioner relationship, and AI accuracy across different ethnic groups, highlighting the necessity for targeted education and transparent communication to build trust for successful AI integration [28].

## What are the main strengths and weaknesses?

A key strength of the NELDS cohort is its rich ethnic diversity. Only 36% self-reported as having White ethnicity, with substantial proportions from South Asian (34%) and Black (16%) communities. This broad ethnic representation is particularly pertinent given that the rates of diabetes are notably higher among Black and South Asian groups in England compared with White groups [29].

This contrasts sharply with other major cohorts, including the Scottish Diabetes Research Network National Diabetes Cohort (73.1% White) [30], SCONe (77.5% White) [31], and the Danish DD2 and Swedish ANDIS cohorts, which are presumably ethnically homogeneous [32, 33]. While the Singapore SEED study offers Asian-population insights, it lacks White and Black representation, and relies on repeat examinations rather than longitudinal EHR data linkage [34].

Furthermore, more than two-thirds of the individuals reside in the two most deprived IMD quintiles, consistently with national trends showing that ethnic-minority groups are disproportionately affected by socio-economic deprivation [35]. This enables the in-depth investigation of socio-economic inequalities in diabetes outcomes and access to care.

The integration of longitudinal ophthalmic imaging data alongside expert DR grading and linked EHRs is a distinguishing feature of the NELDS, positioning the cohort to contribute to the emerging field of oculomics [36]. While large biobank cohorts such as UK Biobank contain some retinal images [37], they lack systematic DR screening data and detailed ophthalmic EHR data, limiting their utility for developing predictive models that incorporate ophthalmic biomarkers. The NELDS also provides a multi-ethnic external validation set for models developed in other cohorts.

Despite its strengths, the NELDS has some limitations. Images and DR gradings from predecessor screening programmes prior to 2012 are not included. As with all large-scale observational studies, there may be missing or conflicting data within routinely collected EHRs, e.g. regarding diabetes type or date of diagnosis. The retrospective capture of Hospital Episode Statistics is limited to records from April 2015 onwards, potentially underestimating the baseline comorbidity prevalence, though this does not affect the prospective capture of incident events. There is also the potential to miss some mortality data due to the timing of data extraction. Finally, detailed outcomes from specialist ophthalmic services may have been incompletely fed back into the DESP. Future work will address this through linkage to tertiary ophthalmic EHR data [38].

## Can I get hold of the data? Where can I find out more?

The NELDS data are not publicly available due to restrictions on data sharing and the highly sensitive nature of the linked, longitudinal records, which include clinical images. The programme is open to considering collaborative work on a case-by-case basis. Approved collaborations requiring access to the data for analysis must be conducted within the secure, approved Trusted Research Environment (TRE) at Homerton Healthcare NHS Foundation Trust, London, UK. Enquiries regarding collaboration opportunities should be directed to the corresponding author.

## Ethics approval

Ethical approval was obtained from the NHS Health Research Authority and Health and Care Research Wales (IRAS project ID 265637). Approval from the head of research governance at the lead clinical centre was obtained. Individual consent forms were not deemed necessary for ethics approval, as the study was confined to the utilization of retrospective anonymized data, stored and analysed within a secure TRE (see Supplementary methods). The study was conducted in accordance with the Declaration of Helsinki.

## Supplementary Material

dyag117_Supplementary_Data

## Use of artificial intelligence (AI) tools

AI-assisted tools were used in a limited capacity during the preparation of this manuscript, restricted to minor rephrasing of the text for clarity and generating short code snippets to support data processing. AI tools did not contribute to the study design, data collection, analysis, interpretation of results, or the intellectual content of this work. All AI-generated outputs were reviewed, verified, and edited by the authors, who take full responsibility for the accuracy and integrity of this manuscript.
