# Supplementary material for: Cohort Profile: The North East London Diabetes Study (NELDS)
Source: Int J Epidemiol. 2026 Aug 5;55(4):dyag117. doi: 10.1093/ije/dyag117 (PMC13440672; doi:10.1093/ije/dyag117)
Supplement: Supplementary file 1 [file dyag117_supplementary_data.zip › ije-2025-10-2004-File002.docx]

# Supplementary Methods

### Diabetic retinopathy screening and grading

In the United Kingdom (UK), annual photographic diabetic eye screening is offered to all people with diabetes aged 12 or older. The National DESP consists of a number of local DESPs which adhere to national standards and guidelines. The NEL DESP is provided by the Homerton Healthcare NHS Foundation Trust, serving a population which has higher than national average levels of deprivation and a varied health profile with lower than average life expectancy,^1^ spanning the boroughs of Newham, Redbridge, Tower Hamlets and Waltham Forest, classified as the most ethnically diverse in London; and the boroughs of Hackney, Havering, and Barking and Dagenham, which also have a substantial multiethnic population.^2,3^

The NEL DESP adheres to English NHS DESP standards.^4^ People with diabetes aged ≥12 years are identified through the electronic ‘General Practice to Diabetic Retinopathy Screening’ coding system, which automatically notifies DESPs about new diabetes diagnoses. All eligible people are invited for screening within 3 months of notification. Over the course of 1 year, every person eligible for diabetic eye screening is offered multiple opportunities to attend if appointments are missed or cancelled.^5,6^ Since October 2023, people attending the DESP with two consecutive screening episodes with no evidence of DR in both eyes are eligible for screening appointments every 2 years.^7^ The Homerton Healthcare carries out appointment call/recall, screening, image grading, referral tasks, and is responsible for providing clinical leadership and programme management, including failsafe procedures and internal quality assurance.^1,8^

As we have described previously,^5^ a screening visit entails history taking by specialist staff, visual acuity assessment, and capture under pupil dilation of two 45° digital retinal images, centred on the fovea and optic nerve respectively, for each eye. Trained graders assess the images for presence and severity of diabetic retinopathy following a multilevel internally and externally quality-assured process.^4^ Diabetic retinopathy outcomes are recorded as per the UK National Screening Committee classification system (NSC-UK).^9^ Grades in order of increasing severity are: no retinopathy (R0), mild non-proliferative diabetic retinopathy (R1), severe non-proliferative diabetic retinopathy (R2), diabetic maculopathy (M1), and proliferative diabetic retinopathy (R3). Sight-threatening diabetic retinopathy (or referable diabetic retinopathy) comprises retinopathy grades greater than or equal to R2 and or presence of diabetic maculopathy (M1), warranting referral to hospital eye services for further assessment and treatment.

### Diabetic Eye Screening data

People invited to attend diabetic eye screening appointments within the study period were identified and an anonymised data extraction for all available appointments was completed by RC using structured query language searches. A postcode-derived index of multiple deprivation (IMD) rank score was calculated for each episode at data source and only the rank scores were extracted for creation of the final database.

Routinely collected data from DES included age at first appointment, sex, self-defined ethnicity (coded as per Office for National Statistics standards as: white, black, South Asian, Chinese, any other Asian, mixed, other, and unknown categories for the purpose of these analyses),^10^ self-defined type of diabetes (type 2, type 1, and other), self-defined duration of diabetes, visual acuity, diabetic retinopathy severity, and IMD. The IMD combines and weights indicators of deprivation and is the nationally recognised measure of relative deprivation in England.^11^ IMD scores were split into quintiles (where first and fifth are the most and least deprived, respectively) in accordance with 2019 English indices of deprivation.^11^ A database was created by RC as a comma delimited file.

Two-field retinal imaging for each eye is obtained in every visit (minimum of two images per eye) using non-mydriatic approved fundus cameras as per NHS standards.^12^ As per routine screening practice, additional images can be obtained to ensure images of sufficient quality for grading or to document pathology, including anterior segment imaging or non-ocular images (e.g. a hand or tissue box) as evidence of camera functionality when a patient cannot be imaged. Images and metadata were recorded in an OptoMize (NEC Software Solutions UK Limited) database in the Homerton Healthcare NEL DESP. An anonymised extraction of all retinal images with metadata including time-stamped capture data was stored as a separate database.

### Electronic health record data

EHR data from primary and secondary care were extracted for the cohort by the NHS Discovery Data Service, linking by NHS number. The secondary care data (Hospital Episode Statistics) are available from 1 April 2015, due to legal and Information Governance constraints on the retrospective extract. This included all GP prescription (SNOMED CT) and Hospital Episode Statistics data (diagnoses, ICD-10; operations, OPCS-4), as well as GP diagnostic and investigations records for a curated subset of SNOMED CT codes in order to comply with GDPR regulations. For the latter dataset, we included all SNOMED CT codes that map to ICD-10 and OPCS-4, codes that form code lists published on the HDR UK Human Phenotype Library website,^13^ Quality and Outcomes Framework codes,^14^ and a further set of manually curated diabetes-related codes. Mapping tables from SNOMED CT to ICD-10 and OPCS-4 were obtained from the SNOMED CT UK Monolith Edition, RF2: Snapshot Release 35.4.0, available from the NHS Technology Reference Data Update Distribution (TRUD) website.^15^ Read 2 and Read 3 codelists from the HDR UK Human Phenotype Library were mapped to SNOMED CT using mapping tables from the NHS Data Migration Release 29.0.0, also available from the NHS TRUD website. The complete final list of included SNOMED CT codes and numbers of EHR entries is available in the Supplementary Excel.

### Trusted Research Environment

A designated Trusted Research Environment (TRE) was specified and set up within the secure network of Homerton Healthcare to host the hardware and software necessary for data storage and analysis. The required approvals for TRE creation, in accordance with the Data Sharing Framework in North East London, were obtained. A Data Protection Impact Assessment (DPIA) form was submitted and approved by the NHS North East London Information Governance Steering Group (IGSG) and the Data Access Group. Further details on TRE set up and technical specifications can be found in the publication by Fajtl and colleagues.^16^ Access to the TRE is restricted to approved researchers with Homerton Healthcare contracts. Data transfers to and from the TRE are restricted to the data manager (RC), with all transfers requiring approval from the data guardian (JVA).

### Characterisation at baseline

For the purposes of the present paper baseline was defined for each patient as the date of their first completed NEL DESP screening episode during the study period. Diabetes-related comorbidities, medications, and physical and biochemical measurements were identified from both primary (SNOMED CT) and secondary care (ICD-10, diagnoses; OPCS-4, procedures) records using QOF codelists for comorbidities, where available, and otherwise manually curated codelists (AW and AO-B) using the codeminer R package.^17^ Equivalent ICD-10 and OPCS-4 codes for QOF codelists (SNOMED CT) were created by mapping QOF codelists to ICD-10 and OPCS-4 (note, not all QOF codelists mapped to OPCS-4), followed by manual review (AW and AO-B). Medication codelists were furthermore reviewed by clinical pharmacologists (AH and RS). All codelists are provided in the Supplementary Excel.

Comorbidities (systemic arterial hypertension, myocardial infarction, stroke and chronic kidney disease) and medication types (anti-hypertensives, lipid-lowering, non-insulin hypoglycaemic and insulin medications) were defined by the presence of any codes from their corresponding codelists in primary or secondary care records, with the earliest record date taken as the date of diagnosis or treatment commencement. Individuals were therefore deemed to have these comorbidities or medication types at baseline if the EHR-derived diagnosis or treatment commencement date was earlier than or equal to the date of their first NEL DESP screening episode.

A range of plausible values for physical and biochemical measurements were defined (Supplementary Excel). Measurements outside the pre-defined range were defined as implausible and removed. For each individual, the mean values of all measurements recorded within 5 years preceding their initial NEL DESP screening episode were utilised for the present analysis.

### Statistics

Baseline characteristics are presented as median (interquartile range) for continuous variables and number (%) for categorical variables. Age-standardised prevalence and incidence rates were calculated based on the WHO 2000-2025 World Standard Population, using direct standardisation and Byar’s approximation with Dobson adjustment for 95% confidence intervals. Analyses were conducted using R version 4.3.2.

## References

1.	Public Health England. Screening Quality Assurance Visit Report - NHS Diabetic Eye Screening Service North East London Diabetic Eye Screening Programme. https://assets.publishing.service.gov.uk/government/uploads/system/uploads/attachment_data/file/657800/North_east_london_qa_visit_diabetic_eye_executive_summary_may_2017.pdf (2017).

2.	Regional ethnic diversity. https://www.ethnicity-facts-figures.service.gov.uk/uk-population-by-ethnicity/national-and-regional-populations/regional-ethnic-diversity/latest/ (2022).

3.	National identity, England and Wales - Office for National Statistics. https://www.ons.gov.uk/peoplepopulationandcommunity/culturalidentity/ethnicity/bulletins/nationalidentityenglandandwales/census2021.

4.	Public Health England. Diabetic eye screening programme: standards. GOV.UK https://www.gov.uk/government/publications/diabetic-eye-screening-programme-standards (2019).

5.	Olvera-Barrios, A. et al. Effect of ethnicity and other sociodemographic factors on attendance at diabetic eye screening: a 12-month retrospective cohort study. BMJ Open **11**, e046264 (2021).

6.	Diabetic eye screening: cohort management. GOV.UK https://www.gov.uk/government/publications/diabetic-eye-screening-cohort-management-overview/diabetic-eye-screening-cohort-management.

7.	Diabetic eye screening intervals extended for people at lowest risk. GOV.UK https://www.gov.uk/government/publications/diabetic-eye-screening-longer-intervals-for-people-at-lowest-risk/diabetic-eye-screening-intervals-extended-for-people-at-lowest-risk.

8.	England, N. H. S. NHS England » NHS Diabetic Eye Screening Programme: screening quality assurance visit reports. https://www.england.nhs.uk/publication/nhs-diabetic-eye-screening-programme-screening-quality-assurance-visit-reports/ (2023).

9.	Harding, S. et al. Grading and disease management in national screening for diabetic retinopathy in England and Wales. Diabet. Med. J. Br. Diabet. Assoc. **20**, 965–971 (2003).

10.	Office for National Statistics. Ethnicity and National Identity in England and Wales - Office for National Statistics. https://www.ons.gov.uk/peoplepopulationandcommunity/culturalidentity/ethnicity/articles/ethnicityandnationalidentityinenglandandwales/2012-12-11#measuring-ethnicity.

11.	Office for National Statistics. English indices of deprivation 2019. GOV.UK https://www.gov.uk/government/statistics/english-indices-of-deprivation-2019.

12.	Diabetic eye screening: approved cameras and settings. GOV.UK https://www.gov.uk/government/publications/diabetic-eye-screening-approved-cameras-and-settings (2025).

13.	HDRUK Phenotype Library. http://phenotypes.healthdatagateway.org/.

14.	NHS England Digital. Primary Care Domain Reference Set Portal. NHS England Digital https://digital.nhs.uk/data-and-information/data-collections-and-data-sets/data-collections/quality-and-outcomes-framework-qof/quality-and-outcome-framework-qof-business-rules/primary-care-domain-reference-set-portal (2024).

15.	NHS TRUD. https://isd.digital.nhs.uk/trud/users/guest/filters/0/home.

16.	Fajtl, J. et al. Trustworthy Evaluation of Clinical AI for Analysis of Medical Images in Diverse Populations. NEJM AI **1**, AIoa2400353 (2024).

17.	Warwick, A. Codeminer: An R Package for Clinical Codelist Development. https://github.com/codeminer-io/codeminer (2025).
