# Supplementary material for: Cohort Profile: The North East London Diabetes Study (NELDS)
Source: Int J Epidemiol. 2026 Aug 5;55(4):dyag117. doi: 10.1093/ije/dyag117 (PMC13440672; doi:10.1093/ije/dyag117)
Supplement: Supplementary file 1 [file dyag117_supplementary_data.zip › ije-2025-10-2004-File004.docx]

## Supplementary Figures

**sFigure 1** Venn diagrams for diabetes-associated comorbidities showing the relative proportions of cases ascertained from primary care (General Practitioner, GP; SNOMED-CT) and secondary care (Hospital Episode Statistics; ICD-10 for diagnoses and OPCS-4 for procedures). Abbreviations: GP = general practitioner; HES = Hospital Episode Statistics; ICD-10 = International Statistical Classification of Diseases and Related Health Problems 10th Revision; OPCS4 = OPCS Classification of Interventions and Procedures version 4.

*Alt text:* Venn diagrams for each diabetes-associated comorbidity showing overlap between primary care and secondary care case ascertainment, with the majority of cases for all conditions identified from primary care records.

## Supplementary Tables

**sTable 1.** Summary of sociodemographic factors at baseline Diabetic Eye Screening Programme visit. Results are presented for the full cohort (shaded grey) and by ethnicity. Abbreviations: CVD = cardiovascular disease; IMD = index of multiple deprivation.

| **Full cohort** | **Ethnicity** | | | | | | | |  |
| --- | --- | --- | --- | --- | --- | --- | --- | --- | --- |
| **Characteristic** | **N = 178,536** | **White N = 64,592** | **South Asian N = 61,202** | **Black N = 29,103** | **Any other Asian N = 9,838** | **Other N = 5,394** | **Mixed N = 2,384** | **Chinese N = 1,190** | **Unknown N = 4,833** |
| **Age (years)** |  |  |  |  |  |  |  |  |  |
| Median (Q1, Q3) | 57 (47, 68) | 62 (51, 72) | 53 (43, 62) | 56 (48, 67) | 54 (45, 63) | 55 (46, 65) | 54 (45, 65) | 59 (49, 67) | 65 (49, 78) |
| **Sex** |  |  |  |  |  |  |  |  |  |
| Female | 81,714 (46%) | 28,258 (44%) | 28,018 (46%) | 14,850 (51%) | 4,389 (45%) | 2,343 (43%) | 1,188 (50%) | 592 (50%) | 2,076 (43%) |
| Male | 96,796 (54%) | 36,327 (56%) | 33,184 (54%) | 14,250 (49%) | 5,449 (55%) | 3,050 (57%) | 1,196 (50%) | 598 (50%) | 2,742 (57%) |
| Unknown | 26 (<0.1%) | 7 (<0.1%) | 0 (0%) | 3 (<0.1%) | 0 (0%) | 1 (<0.1%) | 0 (0%) | 0 (0%) | 15 (0.3%) |
| **Diabetes duration (years)** |  |  |  |  |  |  |  |  |  |
| Median (Q1, Q3) | 2 (0, 8) | 2 (0, 8) | 2 (0, 8) | 2 (0, 8) | 2 (0, 8) | 2 (0, 8) | 1 (0, 5) | 2 (0, 7) | 4 (0, 10) |
| N missing (%) | 4,259 (2.4%) | 1,971 (3.1%) | 378 (0.6%) | 315 (1.1%) | 87 (0.9%) | 49 (0.9%) | 31 (1.3%) | 14 (1.2%) | 1,414 (29%) |
| **Age at diabetes diagnosis (years)** |  |  |  |  |  |  |  |  |  |
| Median (Q1, Q3) | 52 (42, 62) | 56 (46, 67) | 47 (39, 57) | 51 (43, 60) | 49 (41, 58) | 50 (41, 59) | 51 (41, 61) | 55 (45, 62) | 54 (39, 67) |
| N missing (%) | 4,259 (2.4%) | 1,971 (3.1%) | 378 (0.6%) | 315 (1.1%) | 87 (0.9%) | 49 (0.9%) | 31 (1.3%) | 14 (1.2%) | 1,414 (29%) |
| **Died** | 26,547 (15%) | 13,363 (21%) | 5,542 (9.1%) | 3,529 (12%) | 807 (8.2%) | 590 (11%) | 248 (10%) | 109 (9.2%) | 2,359 (49%) |
| **Age at death (years)** |  |  |  |  |  |  |  |  |  |
| Median (Q1, Q3) | 79 (70, 86) | 80 (71, 87) | 77 (67, 83) | 79 (68, 85) | 75 (66, 84) | 76 (67, 84) | 77 (64, 85) | 79 (70, 86) | 79 (71, 85) |
| **IMD quintile** |  |  |  |  |  |  |  |  |  |
| 1 | 61,097 (34%) | 18,428 (29%) | 22,082 (36%) | 13,365 (46%) | 2,539 (26%) | 2,085 (39%) | 855 (36%) | 382 (32%) | 1,361 (28%) |
| 2 | 58,135 (33%) | 19,270 (30%) | 20,796 (34%) | 10,426 (36%) | 3,415 (35%) | 1,928 (36%) | 786 (33%) | 386 (32%) | 1,128 (23%) |
| 3 | 30,395 (17%) | 9,963 (15%) | 12,374 (20%) | 3,488 (12%) | 2,476 (25%) | 905 (17%) | 420 (18%) | 185 (16%) | 584 (12%) |
| 4 | 13,280 (7.4%) | 6,129 (9.5%) | 4,188 (6.8%) | 1,010 (3.5%) | 1,057 (11%) | 284 (5.3%) | 158 (6.6%) | 118 (9.9%) | 336 (7.0%) |
| 5 | 4,281 (2.4%) | 2,698 (4.2%) | 898 (1.5%) | 195 (0.7%) | 173 (1.8%) | 73 (1.4%) | 52 (2.2%) | 61 (5.1%) | 131 (2.7%) |
| Unknown | 11,348 (6.4%) | 8,104 (13%) | 864 (1.4%) | 619 (2.1%) | 178 (1.8%) | 119 (2.2%) | 113 (4.7%) | 58 (4.9%) | 1,293 (27%) |
| **Smoking history** |  |  |  |  |  |  |  |  |  |
| Ever smoked | 52,758 (30%) | 28,205 (44%) | 12,701 (21%) | 6,532 (22%) | 2,047 (21%) | 1,832 (34%) | 709 (30%) | 265 (22%) | 467 (9.7%) |
| Never smoked | 100,000 (56%) | 25,138 (39%) | 43,060 (70%) | 19,123 (66%) | 6,853 (70%) | 2,863 (53%) | 1,402 (59%) | 794 (67%) | 767 (16%) |
| Unknown | 25,778 (14%) | 11,249 (17%) | 5,441 (8.9%) | 3,448 (12%) | 938 (9.5%) | 699 (13%) | 273 (11%) | 131 (11%) | 3,599 (74%) |
| **Family history of CVD** | 66,315 (37%) | 25,041 (39%) | 24,606 (40%) | 8,239 (28%) | 3,764 (38%) | 2,147 (40%) | 851 (36%) | 332 (28%) | 1,335 (28%) |
| **Diabetes type** |  |  |  |  |  |  |  |  |  |
| Type 2 | 163,393 (92%) | 56,533 (88%) | 58,880 (96%) | 27,269 (94%) | 9,457 (96%) | 4,974 (92%) | 2,093 (88%) | 1,114 (94%) | 3,073 (64%) |
| Type 1 | 8,148 (4.6%) | 5,093 (7.9%) | 1,109 (1.8%) | 960 (3.3%) | 190 (1.9%) | 255 (4.7%) | 197 (8.3%) | 41 (3.4%) | 303 (6.3%) |
| Other* | 6,995 (3.9%) | 2,966 (4.6%) | 1,213 (2.0%) | 874 (3.0%) | 191 (1.9%) | 165 (3.1%) | 94 (3.9%) | 35 (2.9%) | 1,457 (30%) |

*’Other’ includes individuals for whom type of diabetes was recorded as ‘Maturity Onset of the Young’, ‘Other’ or ‘Unknown’.

Findings are presented as median (interquartile range) for continuous variables and number (%) for categorical variables.

**sTable 2.** Baseline characteristics of the cohort stratified by permanent loss to follow-up (death or moving out of area) by the end of 2021. Abbreviations: CVD = cardiovascular disease; IMD = index of multiple deprivation.

|  | **Full cohort** | **Died or moved out of area** | |
| --- | --- | --- | --- |
| **Characteristic** | **N = 178,536** | **No N = 134,564** | **Yes N = 43,972** |
| **Age (years)** |  |  |  |
| Median (Q1, Q3) | 57 (47, 68) | 55 (45, 64) | 66 (53, 77) |
| **Sex** |  |  |  |
| Female | 81,714 (46%) | 62,926 (47%) | 18,788 (43%) |
| Male | 96,796 (54%) | 71,638 (53%) | 25,158 (57%) |
| Unknown | 26 (<0.1%) | 0 (0%) | 26 (<0.1%) |
| **Diabetes type** |  |  |  |
| Type 2 | 163,393 (92%) | 125,590 (93%) | 37,803 (86%) |
| Type 1 | 8,148 (4.6%) | 5,653 (4.2%) | 2,495 (5.7%) |
| Other | 6,995 (3.9%) | 3,321 (2.5%) | 3,674 (8.4%) |
| **Diabetes duration (years)** |  |  |  |
| Median (Q1, Q3) | 2 (0, 8) | 1 (0, 7) | 5 (1, 12) |
| N missing (%) | 4,259 (2.4%) | 1,275 (0.9%) | 2,984 (6.8%) |
| **Age at diabetes diagnosis (years)** |  |  |  |
| Median (Q1, Q3) | 52 (42, 62) | 50 (41, 60) | 57 (45, 68) |
| N missing (%) | 4,259 (2.4%) | 1,275 (0.9%) | 2,984 (6.8%) |
| **Ethnicity** |  |  |  |
| White | 64,592 (36%) | 43,925 (33%) | 20,667 (47%) |
| South Asian | 61,202 (34%) | 51,829 (39%) | 9,373 (21%) |
| Black | 29,103 (16%) | 22,942 (17%) | 6,161 (14%) |
| Any other Asian | 9,838 (5.5%) | 8,079 (6.0%) | 1,759 (4.0%) |
| Other | 5,394 (3.0%) | 4,241 (3.2%) | 1,153 (2.6%) |
| Unknown | 4,833 (2.7%) | 708 (0.5%) | 4,125 (9.4%) |
| Mixed | 2,384 (1.3%) | 1,881 (1.4%) | 503 (1.1%) |
| Chinese | 1,190 (0.7%) | 959 (0.7%) | 231 (0.5%) |
| **IMD quintile** |  |  |  |
| 1 | 61,097 (34%) | 46,888 (35%) | 14,209 (32%) |
| 2 | 58,135 (33%) | 44,275 (33%) | 13,860 (32%) |
| 3 | 30,395 (17%) | 23,332 (17%) | 7,063 (16%) |
| 4 | 13,280 (7.4%) | 10,129 (7.5%) | 3,151 (7.2%) |
| 5 | 4,281 (2.4%) | 3,159 (2.3%) | 1,122 (2.6%) |
| Unknown | 11,348 (6.4%) | 6,781 (5.0%) | 4,567 (10%) |
| **Smoking history** |  |  |  |
| Ever smoked | 52,758 (30%) | 41,939 (31%) | 10,819 (25%) |
| Never smoked | 100,000 (56%) | 85,520 (64%) | 14,480 (33%) |
| Unknown | 25,778 (14%) | 7,105 (5.3%) | 18,673 (42%) |
| **Family history of CVD** | 66,315 (37%) | 52,977 (39%) | 13,338 (30%) |
| *’Other’ includes individuals for whom type of diabetes was recorded as ‘Maturity Onset of the Young’, ‘Other’ or ‘Unknown’.  Findings are presented as median (interquartile range) for continuous variables and number (%) for categorical variables. Diabetic retinopathy outcomes are recorded as per the UK National Screening Committee classification system (Supplementary methods). | | | |

**sTable 3.** Number and percentage of individuals with at least one entry across the full study period for specific clinical measures in their primary care records, by diabetes subtype. Abbreviations: BMI = body mass index; eGFR = estimated glomerular filtration rate; HbA1c = haemoglobin A1C.

|  | **Full cohort (n = 178,536)** | **Type 2 (n = 163,393)** | **Type 1 (n = 8,148)** | **Other* (n = 6,995)** |
| --- | --- | --- | --- | --- |
| HbA1c | 175,645 (98.4%) | 161,317 (98.7%) | 7,731 (94.9%) | 6,597 (94.3%) |
| Total cholesterol | 176,545 (98.9%) | 162,226 (99.3%) | 7,613 (93.4%) | 6,706 (95.9%) |
| Systolic blood pressure | 177,330 (99.3%) | 162,502 (99.5%) | 7,991 (98.1%) | 6,837 (97.7%) |
| eGFR | 170,042 (95.2%) | 157,166 (96.2%) | 6,881 (84.5%) | 5,995 (85.7%) |
| BMI | 176,618 (98.9%) | 161,954 (99.1%) | 7,896 (96.9%) | 6,768 (96.8%) |

*’Other’ includes individuals for whom type of diabetes was recorded as ‘Maturity Onset of the Young’, ‘Other’ or ‘Unknown’.

Findings are presented as number (%). Diabetic retinopathy outcomes are recorded as per the UK National Screening Committee classification system (Supplementary methods).

**sTable 4.** Summary of clinical measurements and medication history at baseline Diabetic Eye Screening Programme visit. Results are presented for the full cohort (shaded grey) and by ethnicity. Abbreviations: BMI = body mass index; eGFR = estimated glomerular filtration rate; HbA1c = haemoglobin A1C; logMAR = logarithm of the minimum angle of resolution; VA = visual acuity.

| **Full cohort** | **Ethnicity** | | | | | | | |  |
| --- | --- | --- | --- | --- | --- | --- | --- | --- | --- |
| **Characteristic** | **N = 178,536** | **White N = 64,592** | **South Asian N = 61,202** | **Black N = 29,103** | **Any other Asian N = 9,838** | **Other N = 5,394** | **Mixed N = 2,384** | **Chinese N = 1,190** | **Unknown N = 4,833** |
| **Systolic blood pressure (mmHg)** |  |  |  |  |  |  |  |  |  |
| Median (Q1, Q3) | 133 (124, 141) | 135 (127, 142) | 129 (121, 138) | 136 (128, 144) | 131 (123, 138) | 132 (124, 140) | 133 (124, 142) | 130 (122, 138) | 134 (125, 142) |
| N missing (%) | 4,283 (2.4%) | 1,535 (2.4%) | 1,280 (2.1%) | 767 (2.6%) | 186 (1.9%) | 135 (2.5%) | 72 (3.0%) | 29 (2.4%) | 279 (5.8%) |
| **BMI (kg/m²)** |  |  |  |  |  |  |  |  |  |
| Median (Q1, Q3) | 29.2 (25.9, 33.5) | 30.9 (27.1, 35.6) | 27.8 (25.1, 31.2) | 30.3 (27.0, 34.4) | 27.3 (24.7, 30.5) | 29.7 (26.4, 33.7) | 29.6 (25.9, 34.3) | 25.9 (23.4, 28.5) | 28.4 (25.0, 32.8) |
| N missing (%) | 8,999 (5.0%) | 3,790 (5.9%) | 2,407 (3.9%) | 1,449 (5.0%) | 414 (4.2%) | 282 (5.2%) | 143 (6.0%) | 56 (4.7%) | 458 (9.5%) |
| **HbA1c (mmol/mol)** |  |  |  |  |  |  |  |  |  |
| Median (Q1, Q3) | 54 (48, 67) | 53 (47, 66) | 54 (48, 67) | 54 (48, 69) | 54 (48, 66) | 54 (48, 68) | 54 (48, 70) | 52 (48, 62) | 55 (47, 69) |
| N missing (%) | 30,437 (17%) | 11,704 (18%) | 9,329 (15%) | 5,099 (18%) | 1,303 (13%) | 906 (17%) | 366 (15%) | 208 (17%) | 1,522 (31%) |
| **eGFR (ml/min/1.73m²)** |  |  |  |  |  |  |  |  |  |
| Median (Q1, Q3) | 74 (60, 87) | 63 (60, 81) | 82 (66, 90) | 71 (60, 82) | 80 (63, 90) | 79 (66, 88) | 74 (60, 86) | 80 (62, 90) | 64 (59, 85) |
| N missing (%) | 37,733 (21%) | 14,693 (23%) | 12,909 (21%) | 5,016 (17%) | 2,224 (23%) | 1,030 (19%) | 564 (24%) | 232 (19%) | 1,065 (22%) |
| **Total cholesterol (mmol/l)** |  |  |  |  |  |  |  |  |  |
| Median (Q1, Q3) | 4.59 (3.97, 5.27) | 4.60 (4.00, 5.30) | 4.53 (3.90, 5.21) | 4.65 (4.05, 5.30) | 4.58 (3.93, 5.25) | 4.68 (4.05, 5.36) | 4.73 (4.10, 5.50) | 4.68 (4.00, 5.33) | 4.40 (3.77, 5.10) |
| N missing (%) | 7,455 (4.2%) | 2,938 (4.5%) | 2,030 (3.3%) | 1,296 (4.5%) | 315 (3.2%) | 228 (4.2%) | 164 (6.9%) | 43 (3.6%) | 441 (9.1%) |
| **Retinopathy grade, worst** |  |  |  |  |  |  |  |  |  |
| R0 | 127,312 (71%) | 46,406 (72%) | 44,407 (73%) | 20,179 (69%) | 6,965 (71%) | 3,703 (69%) | 1,679 (70%) | 865 (73%) | 3,108 (64%) |
| R1 | 41,759 (23%) | 14,921 (23%) | 14,000 (23%) | 7,065 (24%) | 2,386 (24%) | 1,353 (25%) | 533 (22%) | 269 (23%) | 1,232 (25%) |
| R2 | 3,914 (2.2%) | 1,401 (2.2%) | 1,253 (2.0%) | 664 (2.3%) | 206 (2.1%) | 148 (2.7%) | 62 (2.6%) | 15 (1.3%) | 165 (3.4%) |
| R3 | 2,270 (1.3%) | 764 (1.2%) | 681 (1.1%) | 443 (1.5%) | 127 (1.3%) | 97 (1.8%) | 57 (2.4%) | 11 (0.9%) | 90 (1.9%) |
| Unknown | 3,281 (1.8%) | 1,100 (1.7%) | 861 (1.4%) | 752 (2.6%) | 154 (1.6%) | 93 (1.7%) | 53 (2.2%) | 30 (2.5%) | 238 (4.9%) |
| **Maculopathy grade, worst** |  |  |  |  |  |  |  |  |  |
| M0 | 163,274 (91%) | 59,893 (93%) | 56,254 (92%) | 25,800 (89%) | 8,973 (91%) | 4,869 (90%) | 2,138 (90%) | 1,116 (94%) | 4,231 (88%) |
| M1 | 11,981 (6.7%) | 3,599 (5.6%) | 4,087 (6.7%) | 2,551 (8.8%) | 711 (7.2%) | 432 (8.0%) | 193 (8.1%) | 44 (3.7%) | 364 (7.5%) |
| Unknown | 3,281 (1.8%) | 1,100 (1.7%) | 861 (1.4%) | 752 (2.6%) | 154 (1.6%) | 93 (1.7%) | 53 (2.2%) | 30 (2.5%) | 238 (4.9%) |
| **Best VA (LogMAR)** |  |  |  |  |  |  |  |  |  |
| Median (Q1, Q3) | 0.00 (-0.10, 0.20) | 0.00 (-0.10, 0.20) | 0.00 (-0.10, 0.20) | 0.00 (-0.10, 0.20) | 0.00 (-0.10, 0.20) | 0.00 (-0.10, 0.20) | 0.00 (-0.10, 0.20) | 0.00 (0.00, 0.20) | 0.00 (0.00, 0.20) |
| N missing (%) | 1,570 (0.9%) | 531 (0.8%) | 486 (0.8%) | 294 (1.0%) | 65 (0.7%) | 35 (0.6%) | 16 (0.7%) | 11 (0.9%) | 132 (2.7%) |
| **Anti-hypertension medication** | 112,376 (63%) | 44,306 (69%) | 34,926 (57%) | 18,729 (64%) | 6,052 (62%) | 3,202 (59%) | 1,345 (56%) | 620 (52%) | 3,196 (66%) |
| **Lipid-lowering medication** | 109,465 (61%) | 41,404 (64%) | 37,791 (62%) | 16,103 (55%) | 6,194 (63%) | 3,167 (59%) | 1,240 (52%) | 641 (54%) | 2,925 (61%) |
| **Non-insulin hypoglycaemic medication** | 132,967 (74%) | 45,235 (70%) | 47,931 (78%) | 22,120 (76%) | 7,785 (79%) | 4,044 (75%) | 1,683 (71%) | 832 (70%) | 3,337 (69%) |
| **On insulin** | 27,973 (16%) | 11,378 (18%) | 7,952 (13%) | 5,054 (17%) | 1,094 (11%) | 827 (15%) | 441 (18%) | 122 (10%) | 1,105 (23%) |

Findings are presented as median (interquartile range) for continuous variables and number (%) for categorical variables. Diabetic retinopathy outcomes are recorded as per the UK National Screening Committee classification system (Supplementary methods).

**sTable 5.** Summary of clinical measurements and medication history at baseline Diabetic Eye Screening Programme visit. Results are presented for the full cohort (shaded grey) and by baseline diabetic retinopathy status. Abbreviations: BMI = body mass index; eGFR = estimated glomerular filtration rate; HbA1c = haemoglobin A1C; logMAR = logarithm of the minimum angle of resolution; VA = visual acuity.

| **Full cohort** | **Diabetic retinopathy grade** | | | | |  |
| --- | --- | --- | --- | --- | --- | --- |
| **Characteristic** | **N = 178,536** | **R0 N = 127,312** | **R1 N = 41,759** | **R2 N = 3,914** | **R3 N = 2,270** | **Unknown N = 3,281** |
| **Systolic blood pressure (mmHg)** |  |  |  |  |  |  |
| Median (Q1, Q3) | 133 (124, 141) | 132 (124, 140) | 134 (126, 142) | 137 (128, 145) | 137 (129, 147) | 135 (127, 143) |
| N missing (%) | 4,283 (2.4%) | 2,880 (2.3%) | 1,085 (2.6%) | 131 (3.3%) | 86 (3.8%) | 101 (3.1%) |
| **BMI (kg/m²)** |  |  |  |  |  |  |
| Median (Q1, Q3) | 29.2 (25.9, 33.5) | 29.5 (26.2, 33.8) | 28.6 (25.5, 32.8) | 28.2 (25.1, 32.2) | 28.4 (24.9, 32.5) | 28.1 (24.9, 32.2) |
| N missing (%) | 8,999 (5.0%) | 6,292 (4.9%) | 2,035 (4.9%) | 222 (5.7%) | 165 (7.3%) | 285 (8.7%) |
| **HbA1c (mmol/mol)** |  |  |  |  |  |  |
| Median (Q1, Q3) | 54 (48, 67) | 52 (47, 63) | 59 (50, 75) | 74 (61, 91) | 74 (59, 92) | 54 (47, 67) |
| N missing (%) | 30,437 (17%) | 20,965 (16%) | 7,804 (19%) | 717 (18%) | 351 (15%) | 600 (18%) |
| **eGFR (ml/min/1.73m²)** |  |  |  |  |  |  |
| Median (Q1, Q3) | 74 (60, 87) | 75 (60, 88) | 72 (60, 86) | 67 (60, 84) | 62 (54, 82) | 62 (55, 76) |
| N missing (%) | 37,733 (21%) | 27,113 (21%) | 8,743 (21%) | 842 (22%) | 494 (22%) | 541 (16%) |
| **Total cholesterol (mmol/l)** |  |  |  |  |  |  |
| Median (Q1, Q3) | 4.59 (3.97, 5.27) | 4.65 (4.04, 5.33) | 4.40 (3.80, 5.10) | 4.33 (3.75, 5.03) | 4.42 (3.73, 5.21) | 4.40 (3.77, 5.10) |
| N missing (%) | 7,455 (4.2%) | 5,305 (4.2%) | 1,636 (3.9%) | 193 (4.9%) | 149 (6.6%) | 172 (5.2%) |
| **Maculopathy grade, worst** |  |  |  |  |  |  |
| M0 | 163,274 (91%) | 127,312 (100%) | 33,933 (81%) | 1,218 (31%) | 811 (36%) | 0 (0%) |
| M1 | 11,981 (6.7%) | 0 (0%) | 7,826 (19%) | 2,696 (69%) | 1,459 (64%) | 0 (0%) |
| Unknown | 3,281 (1.8%) | 0 (0%) | 0 (0%) | 0 (0%) | 0 (0%) | 3,281 (100%) |
| **Best VA (LogMAR)** |  |  |  |  |  |  |
| Median (Q1, Q3) | 0.00 (-0.10, 0.20) | 0.00 (-0.10, 0.20) | 0.00 (-0.10, 0.20) | 0.00 (0.00, 0.20) | 0.20 (0.00, 0.30) | 0.20 (0.20, 0.50) |
| N missing (%) | 1,570 (0.9%) | 450 (0.4%) | 133 (0.3%) | 10 (0.3%) | 15 (0.7%) | 962 (29%) |
| **Anti-hypertension medication** | 112,376 (63%) | 76,511 (60%) | 28,584 (68%) | 2,969 (76%) | 1,783 (79%) | 2,529 (77%) |
| **Lipid-lowering medication** | 109,465 (61%) | 74,869 (59%) | 27,947 (67%) | 2,797 (71%) | 1,577 (69%) | 2,275 (69%) |
| **Non-insulin hypoglycaemic medication** | 132,967 (74%) | 92,097 (72%) | 33,351 (80%) | 3,252 (83%) | 1,743 (77%) | 2,524 (77%) |
| **On insulin** | 27,973 (16%) | 12,696 (10.0%) | 11,185 (27%) | 2,064 (53%) | 1,360 (60%) | 668 (20%) |

Findings are presented as median (interquartile range) for continuous variables and number (%) for categorical variables. Diabetic retinopathy outcomes are recorded as per the UK National Screening Committee classification system (Supplementary methods).

**sTable 6.** Summary of sociodemographic factors at baseline Diabetic Eye Screening Programme visit. Results are presented for the full cohort (shaded grey) and by baseline diabetic retinopathy status. Abbreviations: CVD = cardiovascular disease; IMD = index of multiple deprivation.

| **Full cohort** | **Diabetic retinopathy grade** | | | | |  |
| --- | --- | --- | --- | --- | --- | --- |
| **Characteristic** | **N = 178,536** | **R0 N = 127,312** | **R1 N = 41,759** | **R2 N = 3,914** | **R3 N = 2,270** | **Unknown N = 3,281** |
| **Age (years)** |  |  |  |  |  |  |
| Median (Q1, Q3) | 57 (47, 68) | 56 (46, 67) | 58 (48, 69) | 58 (49, 68) | 57 (48, 67) | 72 (60, 80) |
| **Sex** |  |  |  |  |  |  |
| Female | 81,714 (46%) | 60,183 (47%) | 17,506 (42%) | 1,367 (35%) | 864 (38%) | 1,794 (55%) |
| Male | 96,796 (54%) | 67,111 (53%) | 24,250 (58%) | 2,546 (65%) | 1,404 (62%) | 1,485 (45%) |
| Unknown | 26 (<0.1%) | 18 (<0.1%) | 3 (<0.1%) | 1 (<0.1%) | 2 (<0.1%) | 2 (<0.1%) |
| **Diabetes type** |  |  |  |  |  |  |
| Type 2 | 163,393 (92%) | 118,477 (93%) | 37,079 (89%) | 3,195 (82%) | 1,735 (76%) | 2,907 (89%) |
| Type 1 | 8,148 (4.6%) | 4,157 (3.3%) | 3,081 (7.4%) | 442 (11%) | 364 (16%) | 104 (3.2%) |
| Other* | 6,995 (3.9%) | 4,678 (3.7%) | 1,599 (3.8%) | 277 (7.1%) | 171 (7.5%) | 270 (8.2%) |
| **Diabetes duration (years)** |  |  |  |  |  |  |
| Median (Q1, Q3) | 2 (0, 8) | 1 (0, 6) | 6 (1, 13) | 12 (5, 20) | 13 (3, 21) | 5 (0, 12) |
| N missing (%) | 4,259 (2.4%) | 2,695 (2.1%) | 1,010 (2.4%) | 183 (4.7%) | 106 (4.7%) | 265 (8.1%) |
| **Age at diabetes diagnosis (years)** |  |  |  |  |  |  |
| Median (Q1, Q3) | 52 (42, 62) | 52 (43, 62) | 49 (40, 59) | 45 (36, 55) | 43 (32, 54) | 63 (51, 73) |
| N missing (%) | 4,259 (2.4%) | 2,695 (2.1%) | 1,010 (2.4%) | 183 (4.7%) | 106 (4.7%) | 265 (8.1%) |
| **Died** | 26,547 (15%) | 15,655 (12%) | 7,810 (19%) | 1,089 (28%) | 651 (29%) | 1,342 (41%) |
| **Age at death (years)** |  |  |  |  |  |  |
| Median (Q1, Q3) | 79 (70, 86) | 79 (70, 86) | 79 (70, 85) | 73 (63, 81) | 70 (61, 80) | 83 (76, 89) |
| **Ethnicity** |  |  |  |  |  |  |
| White | 64,592 (36%) | 46,406 (36%) | 14,921 (36%) | 1,401 (36%) | 764 (34%) | 1,100 (34%) |
| South Asian | 61,202 (34%) | 44,407 (35%) | 14,000 (34%) | 1,253 (32%) | 681 (30%) | 861 (26%) |
| Black | 29,103 (16%) | 20,179 (16%) | 7,065 (17%) | 664 (17%) | 443 (20%) | 752 (23%) |
| Any other Asian | 9,838 (5.5%) | 6,965 (5.5%) | 2,386 (5.7%) | 206 (5.3%) | 127 (5.6%) | 154 (4.7%) |
| Other | 5,394 (3.0%) | 3,703 (2.9%) | 1,353 (3.2%) | 148 (3.8%) | 97 (4.3%) | 93 (2.8%) |
| Mixed | 2,384 (1.3%) | 1,679 (1.3%) | 533 (1.3%) | 62 (1.6%) | 57 (2.5%) | 53 (1.6%) |
| Chinese | 1,190 (0.7%) | 865 (0.7%) | 269 (0.6%) | 15 (0.4%) | 11 (0.5%) | 30 (0.9%) |
| Unknown | 4,833 (2.7%) | 3,108 (2.4%) | 1,232 (3.0%) | 165 (4.2%) | 90 (4.0%) | 238 (7.3%) |
| **IMD quintile** |  |  |  |  |  |  |
| 1 | 61,097 (34%) | 44,095 (35%) | 13,865 (33%) | 1,253 (32%) | 807 (36%) | 1,077 (33%) |
| 2 | 58,135 (33%) | 41,558 (33%) | 13,505 (32%) | 1,300 (33%) | 727 (32%) | 1,045 (32%) |
| 3 | 30,395 (17%) | 21,708 (17%) | 7,102 (17%) | 656 (17%) | 412 (18%) | 517 (16%) |
| 4 | 13,280 (7.4%) | 9,683 (7.6%) | 3,004 (7.2%) | 258 (6.6%) | 144 (6.3%) | 191 (5.8%) |
| 5 | 4,281 (2.4%) | 3,218 (2.5%) | 902 (2.2%) | 71 (1.8%) | 38 (1.7%) | 52 (1.6%) |
| Unknown | 11,348 (6.4%) | 7,050 (5.5%) | 3,381 (8.1%) | 376 (9.6%) | 142 (6.3%) | 399 (12%) |
| **Smoking history** |  |  |  |  |  |  |
| Ever smoked | 52,758 (30%) | 38,646 (30%) | 12,090 (29%) | 896 (23%) | 479 (21%) | 647 (20%) |
| Never smoked | 100,000 (56%) | 73,570 (58%) | 22,314 (53%) | 1,585 (40%) | 958 (42%) | 1,573 (48%) |
| Unknown | 25,778 (14%) | 15,096 (12%) | 7,355 (18%) | 1,433 (37%) | 833 (37%) | 1,061 (32%) |
| **Family history of CVD** | 66,315 (37%) | 48,897 (38%) | 14,545 (35%) | 1,268 (32%) | 708 (31%) | 897 (27%) |

*’Other’ includes individuals for whom type of diabetes was recorded as ‘Maturity Onset of the Young’, ‘Other’ or ‘Unknown’.

Findings are presented as median (interquartile range) for continuous variables and number (%) for categorical variables. Diabetic retinopathy outcomes are recorded as per the UK National Screening Committee classification system (Supplementary methods).

**sTable 7.** Prevalent cases of major comorbidities at baseline Diabetic Eye Screening Programme visit and subsequent incident events, stratified by baseline diabetic retinopathy status. Incidence rates expressed per 1000 person-years. Diabetic retinopathy outcomes are recorded as per the UK National Screening Committee classification system (Supplementary methods).

| **Comorbidity** | **Baseline retinopathy** | **Prevalent cases** | **Crude prevalence** | **Age-Standardised Prevalence* (95% Confidence Interval)** | **N at risk** | **Incident cases** | **Crude Incidence Rate / 1000 person-years** | **Age-Standardised Incidence Rate / 1000 person-years* (95% Confidence Interval)** |
| --- | --- | --- | --- | --- | --- | --- | --- | --- |
| **Coronary heart disease** |  |  |  |  |  |  |  |  |
|  | R0 | 13,748 | 10.8% | 3.5% (3.5, 3.6) | 113,564 | 5,994 | 9.2 | 3.3 (3.2, 3.4) |
|  | R1 | 5,969 | 14.3% | 5.4% (5.3, 5.6) | 35,790 | 2,878 | 12.9 | 5.5 (5.2, 5.7) |
|  | R2 | 667 | 17.0% | 7.3% (6.6, 7.9) | 3,247 | 395 | 20.4 | 10.2 (8.9, 11.5) |
|  | R3 | 391 | 17.2% | 8.1% (7.1, 9.0) | 1,879 | 231 | 24.7 | 13.8 (11.5, 16.1) |
|  | Unknown | 575 | 17.5% | 4.9% (3.7, 6.1) | 2,706 | 182 | 13.1 | 5.4 (3.8, 7.0) |
| **Foot ulcer** |  |  |  |  |  |  |  |  |
|  | R0 | 1,381 | 1.1% | 0.8% (0.7, 0.9) | 125,931 | 1,726 | 2.3 | 1.5 (1.2, 1.8) |
|  | R1 | 778 | 1.9% | 1.7% (1.2, 2.2) | 40,981 | 1,154 | 4.4 | 3.7 (2.1, 5.3) |
|  | R2 | 184 | 4.7% | 4.4% (3.0, 5.8) | 3,730 | 293 | 12.9 | 9.3 (7.5, 11.1) |
|  | R3 | 173 | 7.6% | 5.8% (4.4, 7.2) | 2,097 | 189 | 17.5 | 14.8 (10.2, 19.3) |
|  | Unknown | 109 | 3.3% | 2.0% (0.8, 3.2) | 3,172 | 101 | 6.1 | 4.5 (2.4, 6.5) |
| **Hypertension** |  |  |  |  |  |  |  |  |
|  | R0 | 61,687 | 48.5% | 19.7% (19.5, 20.0) | 65,625 | 12,673 | 40.1 | 20.3 (19.8, 20.8) |
|  | R1 | 22,927 | 54.9% | 26.4% (25.9, 26.9) | 18,832 | 4,314 | 43.6 | 26.1 (24.9, 27.3) |
|  | R2 | 2,311 | 59.0% | 32.2% (30.3, 34.2) | 1,603 | 456 | 57.0 | 38.6 (33.2, 44.0) |
|  | R3 | 1,401 | 61.7% | 36.2% (33.7, 38.8) | 869 | 243 | 66.4 | 53.3 (44.9, 61.7) |
|  | Unknown | 2,130 | 64.9% | 25.2% (22.7, 27.8) | 1,151 | 225 | 43.0 | 24.7 (19.2, 30.1) |
| **Peripheral artery disease** |  |  |  |  |  |  |  |  |
|  | R0 | 1,911 | 1.5% | 0.5% (0.4, 0.5) | 125,401 | 1,703 | 2.3 | 0.9 (0.8, 1.0) |
|  | R1 | 950 | 2.3% | 0.8% (0.8, 0.9) | 40,809 | 1,260 | 4.8 | 2.4 (2.0, 2.8) |
|  | R2 | 168 | 4.3% | 2.0% (1.6, 2.5) | 3,746 | 319 | 13.9 | 8.6 (6.9, 10.3) |
|  | R3 | 134 | 5.9% | 3.3% (2.6, 4.0) | 2,136 | 198 | 18.0 | 12.7 (10.1, 15.3) |
|  | Unknown | 126 | 3.8% | 1.2% (0.4, 2.0) | 3,155 | 100 | 6.1 | 3.2 (1.7, 4.7) |
| **Renal failure** |  |  |  |  |  |  |  |  |
|  | R0 | 14,119 | 11.1% | 3.9% (3.8, 4.0) | 113,193 | 11,244 | 17.9 | 6.8 (6.5, 7.1) |
|  | R1 | 6,708 | 16.1% | 6.3% (6.1, 6.5) | 35,051 | 5,209 | 24.7 | 11.6 (10.8, 12.4) |
|  | R2 | 893 | 22.8% | 12.3% (10.9, 13.7) | 3,021 | 789 | 47.0 | 41.7 (17.6, 65.7) |
|  | R3 | 682 | 30.0% | 17.8% (15.9, 19.7) | 1,588 | 470 | 65.4 | 51.7 (43.9, 59.4) |
|  | Unknown | 887 | 27.0% | 7.1% (5.8, 8.5) | 2,394 | 456 | 38.8 | 13.8 (8.5, 19.0) |
| **Stroke** |  |  |  |  |  |  |  |  |
|  | R0 | 3,386 | 2.7% | 0.9% (0.8, 0.9) | 123,926 | 2,991 | 4.1 | 1.2 (1.1, 1.2) |
|  | R1 | 1,531 | 3.7% | 1.3% (1.3, 1.4) | 40,228 | 1,689 | 6.6 | 2.2 (2.1, 2.3) |
|  | R2 | 205 | 5.2% | 2.2% (1.9, 2.6) | 3,709 | 242 | 10.6 | 4.4 (3.7, 5.2) |
|  | R3 | 168 | 7.4% | 3.5% (2.9, 4.0) | 2,102 | 151 | 13.9 | 7.1 (5.3, 8.9) |
|  | Unknown | 303 | 9.2% | 2.5% (1.8, 3.1) | 2,978 | 158 | 10.1 | 2.8 (1.7, 3.8) |

*Age-standardised prevalence and incidence rates were calculated based on the World Health Organization 2000-2025 World Standard Population.
